# Efficient Room-Temperature Phosphorescence from Discrete Molecules Based on Thianthrene Derivatives for Oxygen Sensing and Detection

**DOI:** 10.3389/fchem.2021.810304

**Published:** 2022-01-27

**Authors:** Zhiqiang Yang, Shuaiqiang Zhao, Xiangyu Zhang, Meng Liu, Haichao Liu, Bing Yang

**Affiliations:** State Key Laboratory of Supramolecular Structure and Materials, College of Chemistry, Jilin University, Changchun, China

**Keywords:** pure organic room-temperature phosphorescence, discrete molecule, thianthrene, ratiometric optical oxygen sensing, polymer film, folding geometry, spin-orbit coupling

## Abstract

In this work, two thianthrene (TA) derivatives, 1-phenylthianthrene (TA1P) and 2-phenylthianthrene (TA2P), were synthesized with single-phenyl modification for pure organic discrete-molecule room-temperature phosphorescence (RTP). They both show the dual emission of fluorescence and RTP in amorphous polymer matrix after deoxygenation, as a result of a new mechanism of folding-induced spin-orbit coupling (SOC) enhancement. Compared with TA1P, TA2P exhibits a higher RTP efficiency and a larger spectral separation between fluorescence and RTP, which is ascribed to the substituent effect of TA at the 2-position. With decreasing oxygen concentration from 1.61% to 0%, the discrete-molecule TA2P shows an about 18-fold increase in RTP intensity and an almost constant fluorescence intensity, which can make TA2P as a self-reference ratiometric optical oxygen sensing probe at low oxygen concentrations. The oxygen quenching constant (*K*_SV_) of TA2P is estimated as high as 10.22 KPa^−1^ for polymethyl methacrylate (PMMA)-doped film, and even reach up to 111.86 KPa^−1^ for Zeonex^®^-doped film, which demonstrates a very high sensitivity in oxygen sensing and detection. This work provides a new idea to design pure organic discrete-molecule RTP materials with high efficiency, and TA derivatives show a potential to be applied in quantitative detection of oxygen as a new-generation optical oxygen-sensing material.

## 1 Introduction

Due to high efficiency and long lifetime of luminescence, phosphorescent materials have received increasing attention, resulting in their broad applications in light-emitting devices (Higginbotham et al., 2021; Wang J. et al., 2021; Zhang et al., 2021), information encryption and storage (Bian et al., 2018; Gu et al., 2018; She et al., 2021), biological imaging (Yang J. et al., 2018; Yang et al., 2021), photodynamic therapy (Shi et al., 2014; Wang et al., 2020), molecular detection and sensing (Yu et al., 2017; Zhou et al., 2019; Liu et al., 2020), etc. Most of the phosphorescent materials are focused on metal complexes [such as Os (Omar et al., 2018), Ir (Idris et al., 2021; Mao et al., 2021), and Pt (Yang X. et al., 2018; Stipurin and Strassner, 2021)], due to the strong spin-orbit coupling (SOC) from the heavy-atom effect of metal atoms. However, they still have some drawbacks from high cost, resource scarcity, and potential toxicity of these noble metals, so the pure organic metal-free room-temperature phosphorescence (RTP) material becomes an ideal alternative. Limited by the weak SOC in pure organic materials, their phosphorescence radiation experiences an extremely slow process (Zhao et al., 2020), which leads to very easy deactivation of triplet excitons by molecular thermal motions or external oxygen quenching. In this case, two pathways are commonly used to obtain the efficient pure organic RTP: one is to promote the generation of triplet excitons by enhancing SOC effect with an introduction of heavy atoms (e.g., Br, I, and Se) (Wen et al., 2019; Lee et al., 2020) or heteroatoms (e.g., O, N, and S) (Xu et al., 2017; Wen et al., 2020; Xu et al., 2020; Wen et al., 2021) in molecular systems; the other one is to suppress the non-radiative decay of triplet excitons by strengthening the system rigidity from crystallization and host–guest interactions (Gu et al., 2019; Qu et al., 2019). However, this crystalline state greatly increases the difficulty of material processing and limits the practical applications of pure organic RTP materials. As a matter of fact, it is more convenient for organic emitters to be widely used in the form of amorphous film comparing with crystalline state, especially for the doped film with discrete molecules in polymer matrix, showing excellent processability. Nevertheless, the efficient discrete-molecule RTP materials in amorphous film have been rarely reported (Li et al., 2018; Ma et al., 2018; Ma et al., 2019; Zhang et al., 2020), because the discrete-molecule RTP in amorphous state frequently encounters a competitive non-radiative decay of triplet excitons. As for the reasons, on the one hand, the environmental rigidity is not large enough to effectively suppress the molecular vibrational quenching; on the other hand, triplet state oxygen can sensitively quench RTP through non-radiative energy transfer *via* molecular collisions under ambient conditions (Lee et al., 2013).

Using this unique oxygen-sensitive property, the pure organic RTP materials can be also applied in the optical oxygen sensing and detection, which involves many aspects of the application, such as physiological and pathological detection (Zhang et al., 2009; Spencer et al., 2014; Zhang et al., 2018), food packaging and storage (Smiddy et al., 2002; Mills, 2005; O’Mahony et al., 2006), and so on. Commonly, the ratiometric measurement method is used to improve the accuracy of optical oxygen detection, which combines an oxygen-insensitive fluorophore and an oxygen-sensitive phosphor into the same polymer matrix, and the oxygen concentration can be quantitatively detected by the ratio of emission intensities between fluorescence and phosphorescence (Feng et al., 2012). For the ratiometric measurement, the metal complex material with single phosphorescence usually needs to be used together with a fluorescence material (Xu et al., 2001), which easily leads to complex problems from multi-components, including sophisticated processing and physical separation (Huang et al., 2018). Thus, the pure organic discrete-molecule RTP material can be a good candidate for ratiometric measurement, because its moderate SOC enables simultaneous and comparable dual-emission between fluorescence and RTP. In addition, such a discrete-molecule dual-emission material is doped in polymer matrix, which surely facilitates the oxygen sensing and detection as a result of good oxygen permeability, unlike the difficult oxygen diffusion in tightly packed molecular crystals (Zhou et al., 2019).

In this work, we designed and synthesized two pure organic compounds, showing the efficient RTP of discrete molecule in polymer film for oxygen sensing and detection. Two compounds, 1-phenylthianthrene (TA1P) and 2-phenylthianthrene (TA2P), were obtained by a simple chemical modification of TA at 1- and 2-positions by using a single-phenyl group. When these two compounds were dispersed in polymethyl methacrylate (PMMA) matrix to form the discrete-molecule-doped film, they exhibit a strong RTP emission band after deoxygenation along with an almost constant fluorescence emission. The efficient RTP emission can be ascribed to the mechanism of folding-induced SOC enhancement (Liu et al., 2018). As a result, the fluorescence intensity can be directly used as a reference signal and RTP intensity can act as an analysis signal, which enable single-component ratiometric optical oxygen sensing and detection without any internal standard substance. Compared with TA1P, the π-conjugation of TA2P is enhanced due to the phenyl substitution at the 2-position of TA, resulting in a larger separation between fluorescence and RTP emission spectra, which substantially decreases the intensity interference between fluorescence and RTP. TA2P demonstrates a better linearity and a larger quenching constant than those of TA1P in oxygen-sensing experiment, which greatly improves the accuracy of oxygen sensing and detection. This work mainly presents a molecular design strategy of the efficient discrete-molecule RTP material, as well as their potential application in terms of oxygen sensing and detection.

## 2 Materials and Methods

### 2.1 Materials and General Methods

All the reagents and solvents used for the synthesis were purchased commercially. The prepared compounds were characterized by ^1^H and ^13^C Nuclear magnetic resonance (NMR) spectra using tetramethylsilane (TMS) as the internal standard (Bruker AVANCE 500 spectrometer), mass spectra (Thermo Fisher ITQ1100 instrument), and elemental analysis (Flash EA 1112, CHNS elemental analysis instrument). UV-vis spectra of films were recorded on a Shimadzu UV-3100 Spectrophotometer. Emission spectra and time-resolved emission spectra were carried out on a FLS980 Spectrometer. Photoluminescence quantum yields under ambient conditions were measured by using an integrating sphere apparatus on a FLS980 Spectrometer, and the excitation wavelength was 300 nm. RTP efficiency was obtained by a comparison between fluorescence and RTP spectra areas under the same experimental conditions. Grayscale images and grayscale values were obtained by using a MATLAB program. The density functional theory (DFT) was adopted for the ground-state geometry optimization at the level of CAM-B3LYP/6-31G(d, p) using Gaussian 09 (version D.01) package (Frisch et al., 2013). The natural transition orbitals (NTOs) were calculated by using time-dependent DFT (TD-DFT) at the level of CAM-B3LYP/6-31G(d, p). SOC coefficients were quantitatively estimated at the level of CAM-B3LYP/6-31G(d, p) by using the Beijing Density Functional (BDF) program (Liu et al., 2003; Li et al., 2012; Quaranta et al., 2012; Li et al., 2013; Li et al., 2014).

### 2.2 Preparation of Film

Firstly, 1 mg TA derivative and 100 mg PMMA particles were weighed, and then both of them were put into a sample tube, followed by a solvent addition of 1.5 ml chloroform; the mixture was left for 30 min until the PMMA was completely dissolved; the mixture of TA derivative and PMMA was further well dispersed by sonicated treatment for 5 min to prepare a homogeneous stock solution. The stock solution was spin-coated on a quartz glass substrate, and a thin film for oxygen sensing and detection was successfully obtained after solvent evaporation and film drying in the air for 30 min.

### 2.3 Synthesis

#### 2.3.1 The Synthesis of 1-Phenylthianthrene

A mixture of 1-thianthrenylboronic acid (520 mg, 2.00 mmol), bromobenzene (471 mg, 3.00 mmol), K_2_CO_3_ (2.48 g, 18.00 mmol), 6 ml distilled water, and 9 ml toluene was degassed and recharged with nitrogen. Then, Pd(PPh_3_)_4_ (69 mg, 0.06 mmol) was added in the mixture as catalyst, and the mixture was degassed and recharged with nitrogen again. After being stirred and refluxed at 90°C for 48 h under nitrogen atmosphere, the mixture was extracted with dichloromethane (DCM). The organic phase was dried with anhydrous sodium sulfate, filtered, and concentrated in vacuum. It was purified *via* silica gel chromatography by the mixture of petroleum ether/DCM and was recrystallized from DCM/methanol to give the product as white powder in 66% yield (385 mg). ^1^H NMR (500 MHz, DMSO-*d*_*6*_, 25°C, TMS): *δ*=7.62 (ddd, *J* = 16.5, 7.7, and 1.4 Hz, 2H), 7.53 (dd, *J* = 8.0 and 6.5 Hz, 2H), 7.50–7.44 (m, 1H), 7.48–7.39 (m, 4H), 7.39–7.32 (m, 2H), and 7.28 (td, *J* = 7.5 and 1.4 Hz, 1H); ^13^C NMR (126 MHz, CDCl_3_-*d*, 25°C, TMS): *δ* = 142.52 (C), 140.21 (C), 136.35 (C), 135.98 (C), 135.40 (C), 135.05 (C), 129.44 (CH), 129.28 (CH), 128.88 (CH), 128.52 (CH), 128.22 (CH), 128.15 (CH), 127.83 (CH), 127.77 (CH), 127.54 (CH), and 127.06 (CH). GC-MS, EI, mass *m*/*z*: 292.42 [M^+^]; anal. calculated for C_12_H_18_S_2_: C 73.94, H 4.14, and S 21.93; found: C 73.97, H 4.20, and S 21.61.

#### 2.3.2 The Synthesis of 2-Phenylthianthrene

According to a previous report, 2-bromothianthrene was synthesized (Liu et al., 2016). TA2P was synthesized by a procedure similar to that of TA1P, and the detail is shown in the Supplementary Material. TA2P product was obtained as white powder in 72% yield (420 mg). ^1^H NMR (500 MHz, DMSO-*d*_*6*_, 25°C, TMS): *δ* = 7.87 (d, *J* = 1.3 Hz, 1H), 7.74–7.69 (m, 2H), 7.66 (d, *J* = 1.2 Hz, 2H), 7.61 (ddd, *J* = 5.5, 3.3, and 1.7 Hz, 2H), 7.48 (t, *J* = 7.6 Hz, 2H), and 7.39 (ddd, *J* = 13.1, 6.6, and 2.3 Hz, 3H); ^13^C NMR (126 MHz, CDCl_3_-*d*, 25°C, TMS): *δ* = 141.09 (C), 139.69 (C), 136.14 (C), 135.57 (C), 135.46 (C), 134.39 (C), 128.91 (CH), 128.81 (CH), 128.76 (CH), 127.75 (CH), 127.28 (CH), 127.00 (CH), and 126.51 (CH). GC-MS, EI, mass *m*/*z*: 292.42 [M^+^]; anal. calculated for C_12_H_18_S_2_: C 73.94, H 4.14, and S 21.93; found: C 74.22, H 4.04, and S 21.02.

## 3 Results and Discussion

**SCHEME 1:**
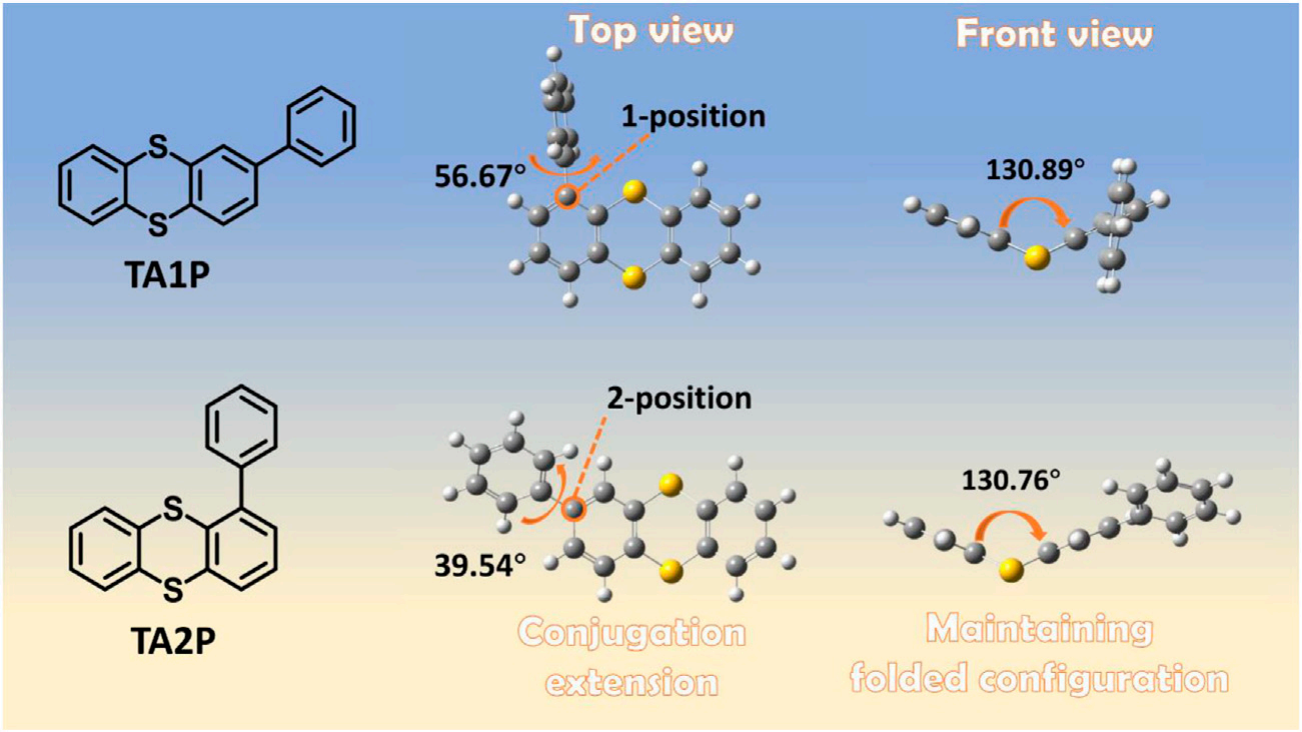
Chemical structures and molecular geometries of TA1P and TA2P.

In our previous work, efficient RTP of TA was reported with a novel mechanism of folding-induced SOC enhancement. TA exhibits not only bright RTP in the crystalline state but also discrete-molecule RTP emission in the doped film of PMMA after deoxygenation. However, under ambient conditions, the discrete-molecule RTP emission cannot be observed in its doped film (Supplementary Figure S1), owing to the complete quenching of triplet state of TA by molecular oxygen in the air. Such a high oxygen sensitivity of TA-doped film inspires us to explore a unique application of TA and its derivatives in oxygen sensing and detection. However, for TA emission, due to the inadequate separation between fluorescence and RTP spectra, the fluorescence intensity is easily affected by the RTP intensity with the decrease of oxygen concentration, which will cause a serious error in ratiometric optical oxygen detection. How to achieve good separation between fluorescence and RTP spectra while maintaining high efficiency of discrete-molecule RTP is a primary focus for molecular design of oxygen-sensitive materials. As regards TA, its high-efficiency discrete-molecule RTP comes from the substantial SOC brought by the folded configuration. Therefore, we can obtain the impressive SOC by maintaining the folded configuration of TA and achieve good separation of fluorescence and RTP spectra by chemical modification of TA. For this reason, TA was further chemically modified at two main sites (1- and 2-position) to design two TA derivatives (TA1P and TA2P), respectively (Scheme 1; Supplementary Scheme S1).

For the oxygen sensing and detection, the polymer matrix is a key factor to determine its working performance. As a priority, PMMA was chosen as the polymer matrix for good film-forming ability, proper oxygen permeability, good UV transmittance, etc. (Wang and Wolfbeis, 2014) The experimental operation process is described in detail in the *Preparation of Film* section (Supplementary Scheme S2).

**FIGURE 1:**
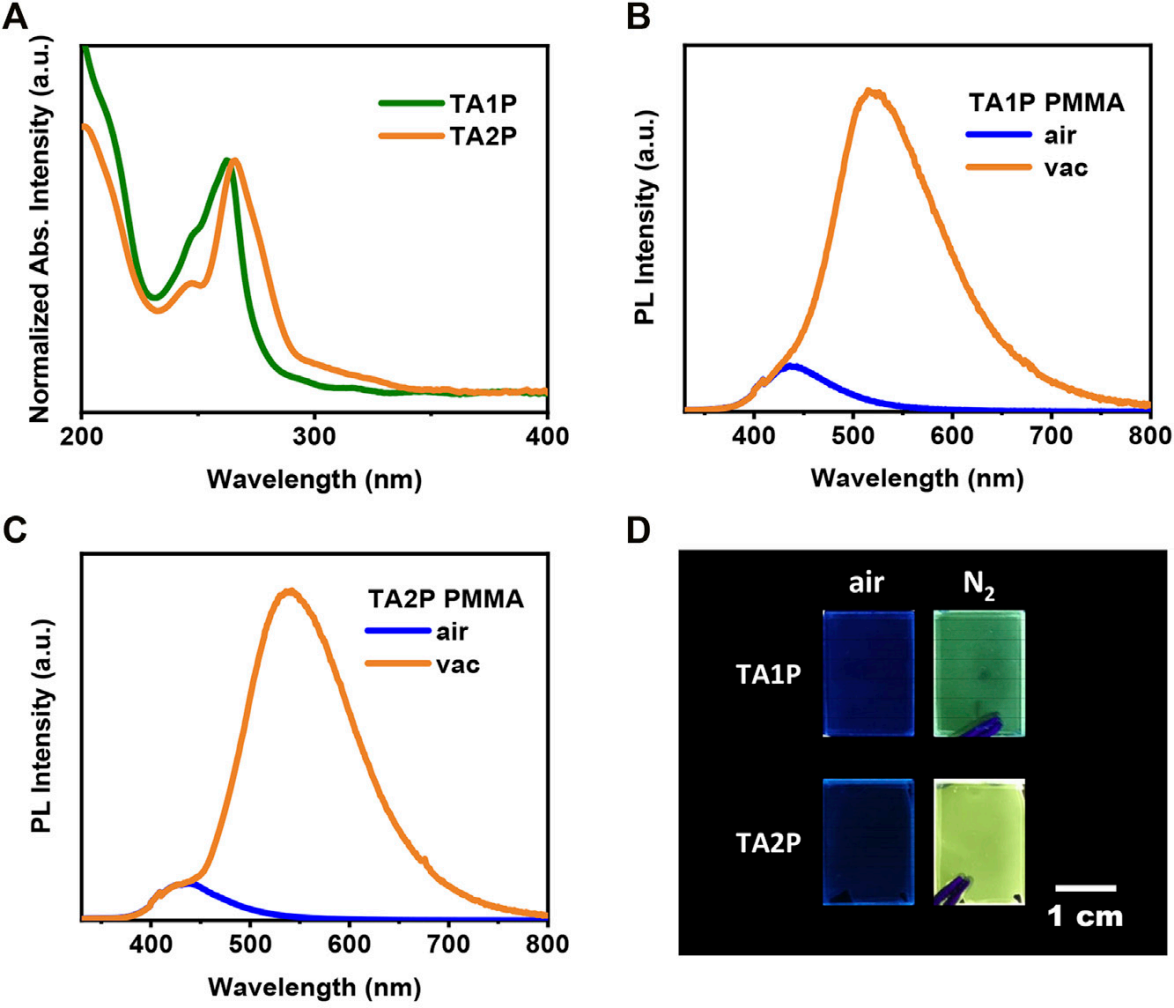
**(A)** UV-vis absorption spectra of 1-phenylthianthrene (TA1P) and 2-phenylthianthrene (TA2P) polymethyl methacrylate (PMMA) films. **(B)**, **(C)** Fluorescence spectra and room-temperature phosphorescence (RTP) spectra of 1 wt.% PMMA-doped films for TA1P and TA2P, respectively. **(D)** Luminous images of TA1P and TA2P PMMA films in the air and purged by nitrogen (N_2_).

Compared with TA, both TA1P and TA2P exhibit different degrees of redshift in their absorption spectra, as a result of the different conjugation effect between phenyl group and TA unit (Figure 1A; Supplementary Figures S2, S3). Obviously, the substitution of phenyl at the 2-position of TA is more effective for conjugation extension than that at the 1-position, and this difference can be clearly understood by theoretical calculations. In the ground state of TA1P (Supplementary Figure S4), the dihedral angle between phenyl group and TA unit is as large as 56.67° due to the steric hindrance. Although the phenyl group participates in the electronic transition of S_0_ → S_1_, the degree of participation is very low, indicating a weak conjugation effect between the phenyl group and TA unit. However, for TA2P (Supplementary Figure S5), the small steric hindrance between phenyl group and TA unit results in a small dihedral angle of 39.54°, and the phenyl group obviously participates in the electronic transition of S_0_ → S_1_, corresponding to an enhanced conjugation. As for emission, both compounds show almost the same blue emission under ambient conditions (the wavelength maximum is at 436 nm in PMMA-doped films). The time-resolved emission spectra demonstrate a short-lived characteristic, indicating the blue fluorescence for the discrete molecules in the doped films (Supplementary Figures S6, S7). As shown in Figures 1B,C, when the doped films are deoxygenated in a vacuum, both materials exhibit a new emission band (peaking at 519 and 533 nm for TA1P and TA2P, respectively) at the long wavelength in addition to the original weak fluorescence emission, and the new emission intensity far exceeds the fluorescence intensity in a vacuum. Time-resolved emission spectra reveal a long-lived characteristic of the long-wavelength emission bands (Supplementary Figure S8), corresponding to the RTP emission. Furthermore, when the film was purged by using a nitrogen stream to simulate the deoxygenation environment, it is obvious that the emission color changes from faint blue to bright green or yellow for these two films (Figure 1D), in good agreement with the spectral measurement in the air and in a vacuum, respectively. Photoluminescence (PL) efficiency is an important performance parameter to evaluate RTP materials for ratiometric optical oxygen sensing and detection. Under ambient conditions, the PL efficiencies of TA1P and TA2P films are measured to be 0.89% and 1.73%, which correspond to their fluorescence efficiencies, respectively. Their RTP efficiencies are estimated to be 8.44% for TA1P and 22.73% for TA2P, which are very high values for pure organic discrete-molecule RTP materials. Compared with TA1P, TA2P has a much higher efficiency of RTP, which is surely more advantageous to be used for oxygen sensing and detection as a detection signal. To understand the origin of RTP of these two materials, the excited states and SOC coefficients were calculated between singlet and triplet states, respectively. Owing to the folding geometry of TA1P and TA2P (the folding dihedral angle is 130.89° and 130.76° for TA units in TA1P and TA2P, respectively), the SOC coefficients are estimated to be very sizeable between singlet and triplet manifolds, which are much higher than those of usual pure organic materials as a result of a new mechanism of folding-induced SOC enhancement. These large SOC coefficients provide very rich intersystem crossing (ISC) channels for the effective generation of triplet excitons, and then the great SOC between T_1_ and S_0_ can ensure the efficient radiation of triplet excitons to produce efficient RTP emission **(**from Supplementary Figures S9–S12).

From qualitative sensing to quantitative detection of oxygen, the detection range should be taken into account firstly. For this reason, a series of nitrogen and oxygen mixtures were prepared with different oxygen concentrations. Considering the complete RTP quenching of discrete-molecule-doped film in the air, the mixed gas should be prepared with an oxygen concentration of less than 21% in later experiments. After careful optimization of conditions, the oxygen concentration range was selected from 0% to 1.61% for subsequent experiments of oxygen detection. For ratiometric oxygen detection, the stability of reference signal is an important factor that affects the accuracy of detection. As shown in Figures 1B,C, the fluorescence intensities of these two films are affected very little, even negligibly, when the RTP intensity is increased dramatically during deoxygenation. This property allows them to be used for single-component ratiometric optical oxygen detection, where the fluorescence emission can directly serve as the reference signal without the addition of an internal standard substance. With increasing oxygen concentration, the fluorescence intensities of the three films (TA, TA1P, and TA2P) were plotted together for the purpose of comparison (Supplementary Figure S13). Among them, the fluorescence intensity of TA2P has the smallest fluctuation with the change of oxygen concentration, demonstrating the best stability of fluorescence intensity as the reference signal for ratiometric optical oxygen detection, which is ascribed to the largest spectral separation between fluorescence (peaking at 436 nm) and RTP (peaking at 533 nm) in essence.

**FIGURE 2:**
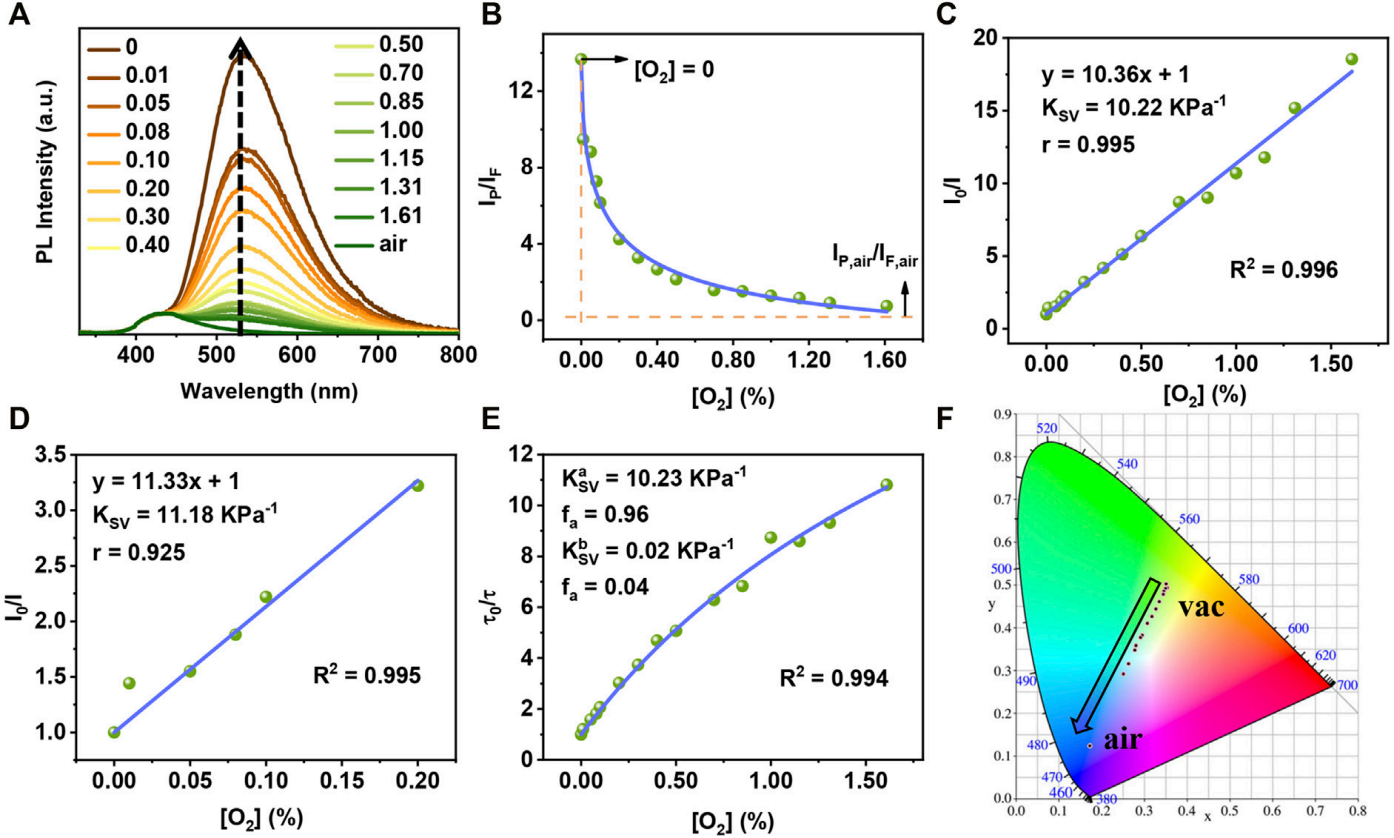
**(A)** Emission spectra of TA2P-PMMA film at different oxygen concentrations (excited by 300 nm). **(B)** The plot of *I*_P_/*I*_F_ against oxygen concentration for TA2P PMMA film. **(C)** Stern–Volmer curve of TA2P PMMA film based on the emission spectrum method (fitting range: 0%–1.61%). **(D)** The Stern–Volmer curve of TA2P PMMA film based on the emission spectrum method (fitting range: 0%–0.20%). **(E)** the “Demas model” curve of TA2P PMMA film based on the lifetime method. **(F)** The Commission Internationale del’Eclairage (CIE) coordinates of TA2P PMMA film with varying oxygen concentrations.

Next, TA2P was taken as an example to carry out oxygen detection experiment. When exposed to the air, the RTP emission of TA2P-doped film is completely quenched, showing pure fluorescence emission. With the decrease of oxygen concentration, the RTP emission is enhanced gradually, while the fluorescence intensity remains almost the same during this process (Figure 2A). For ratiometric optical oxygen detection, a new variable *I* is defined as *I*_P_/*I*_F_, corresponding to a ratio of the RTP emission intensity (*I*_P_) to the fluorescence emission intensity (*I*_F_) at different oxygen concentrations. As shown in Figure 2B, an inverse proportional relation can be obtained for *I* as a function of oxygen concentration. With increasing oxygen concentration, the value of *I* gradually approaches to *I*_P,air_/*I*_F,air_, where *I*_P,air_ and *I*_F,air_ represent the actual spectral intensity at 533 and 436 nm in the air, respectively. When the oxygen concentration is zero, the variable *I* is equal to *I*_0_ in a vacuum. In order to quantitatively evaluate oxygen sensitivity, a linear relationship can be well fitted between *I*_0_/*I* and oxygen concentration according to the Stern–Volmer Eq. 1 (Feng et al., 2012; Wang and Wolfbeis, 2014), as shown in Figure 2C:

$$\frac{I_{0}}{I}=\frac{\tau_{0}}{\tau }=1+K_{SV}\left[O_{2}\right]=1+k_{q}\tau_{0}\left[O_{2}\right]$$ (1)

where *K*_SV_ is the Stern–Volmer quenching constant, *k*_q_ is quenching rate constant, $\left[{\text{O}}_{2}\right]$ is the oxygen concentration, and *τ*_0_ is the RTP lifetime when $\left[{\text{O}}_{2}\right]=0$ .

As a result, the linear fitting of TA2P-doped film shows excellent correlation in the [O_2_] range of 0%–1.61%, and the correlation coefficient (*R* ^2^) is as high as 0.996, corresponding to the Pearson correlation coefficient (Pearson’s *r*) of 0.995. The Stern–Volmer constant (*K*_SV_) is estimated to be 10.22 KPa^−1^, which reflects very high oxygen sensitivity of TA2P film. When the linear fitting was done at an oxygen concentration lower than 0.2%, the correlation coefficient and *K*_SV_ are still as high as 0.994 and 11.18 KPa^−1^, respectively (Figure 2D). This result can guarantee the accuracy of oxygen detection even at a very low oxygen concentration. In the meantime, to verify the dynamic quenching mechanism of oxygen detection, the RTP lifetimes of TA2P film were recorded at different oxygen concentrations. The Stern–Volmer curve of the *τ*_0_/*τ* shows a slight curvature, which is common in most optical oxygen sensors due to the inevitable heterogeneity of materials. In fact, it can be rationalized that there are different micro-zones for the material in different micro-environments. And thus, the quenching constants may be different in different micro-environments, causing a linear deviation of the Stern–Volmer curve. In this case, it can be well described using the “two-site model” by Demas and co-workers, which is also called the “Demas model” Eq. 2 (Carraway et al., 1991; Borisov et al., 2006; Medina-Castillo et al., 2007):

$$\frac{I_{0}}{I}=\frac{\tau_{0}}{\tau }=\frac{1}{\frac{f_{a}}{1+K_{SV}^{a}\left[O_{2}\right]}+\frac{f_{b}}{1+K_{SV}^{b}\left[O_{2}\right]}}$$ (2)

where, ${\text{K}}_{SV}^{a}$ and ${\text{K}}_{SV}^{b}$ are Stern–Volmer constants of different micro-zone components and $f_{a}$ and $f_{b}$ are their corresponding component proportions ( $f_{a}+f_{b}=1$ ), respectively.

As shown in Figure 2E, the Demas model is used for Stern–Volmer curve fitting, resulting in ${\text{K}}_{SV}^{a}$ = 10.23 KPa^−1^ with a large component ratio of 96% (*f*_a_ = 0.96) as well as ${\text{K}}_{SV}^{b}$ = 0.02 KPa^−1^ with a small proportion of only 4% (*f*_b_ = 0.04). Thus, the overall *K*_SV_ can be replaced with ${\text{K}}_{SV}^{a}$ approximatively, and ${\text{K}}_{SV}^{a}$ is almost equal to the *K*_SV_ based on *I*_0_/*I* from Stern–Volmer linear fitting according to Eq. 1 (Supplementary Figure S14), which further validates a dynamic quenching mechanism of RTP in oxygen detection. Besides, the quenching rate constant (*k*_q_) can be calculated as 213.8 KPa^−1^ s^−1^ by Eq. 1, which is one of the highest values among all oxygen-sensing materials (Zhou et al., 2019; Liu et al., 2020; Wang S. et al., 2021). For another key parameter for oxygen detection, the limit of detection (LOD) can be estimated as the oxygen concentration when *I*_P_/*I*_F_ is changed by 0.1% (Guo et al., 2021). For TA2P-doped film, the LOD is calculated to be 0.0979 Pa (equal to 0.966 ppm at atmospheric pressure), which is among one of the best values for oxygen detection.

**FIGURE 3:**
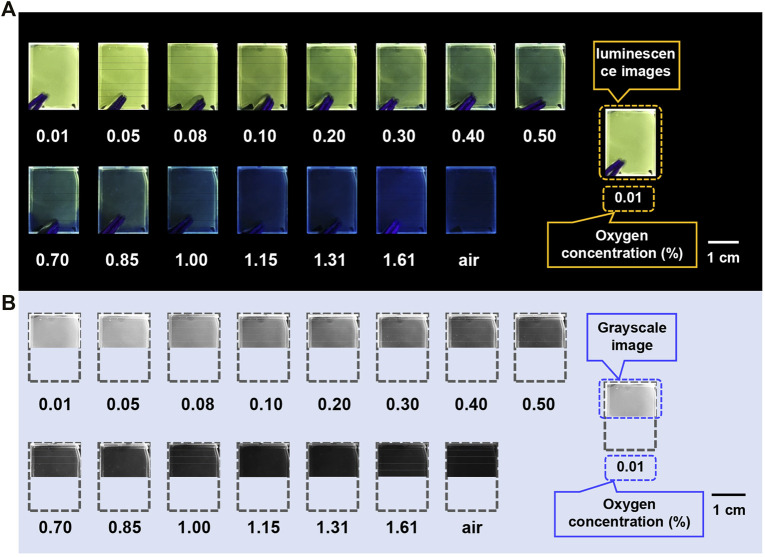
**(A)** Luminescence images of TA2P PMMA-doped films at different oxygen concentrations. **(B)** Grayscale images corresponding to luminescence images at different oxygen concentrations.

Owing to the significant difference between fluorescence and RTP spectra, the emission color change of TA2P can be observed with different oxygen concentrations. This color change can be expressed through the Commission Internationale del’Eclairage (CIE) coordinates, as shown in Figure 2F. As the oxygen concentration gradually increases, the CIE coordinate points move along a line from yellow-green to blue region. Such a linear change gives us an inspiration to conduct the colorimetric detection of oxygen concentration, which is similar to the pH measurement using pH test strips. A simple home-made device was designed to detect the oxygen concentration of nitrogen–oxygen mixed gas stream (Supplementary Figure S15). As shown in Figure 3, when the TA2P film is purged by the mixture of nitrogen and oxygen with different oxygen contents, the color change can be obviously observed with the naked eye. At the same time, the luminous image can be transformed into a grayscale one, and the grayscale value is obtained with the help of computer software (Zhou et al., 2019). In the fitting range of 0.08%–1.15%, a good correlation linearity was found between grayscale value and oxygen concentration (Supplementary Figure S16), which provides a simple method for on-site detection of oxygen concentration in the absence of spectral measurement instrument.

**FIGURE 4:**
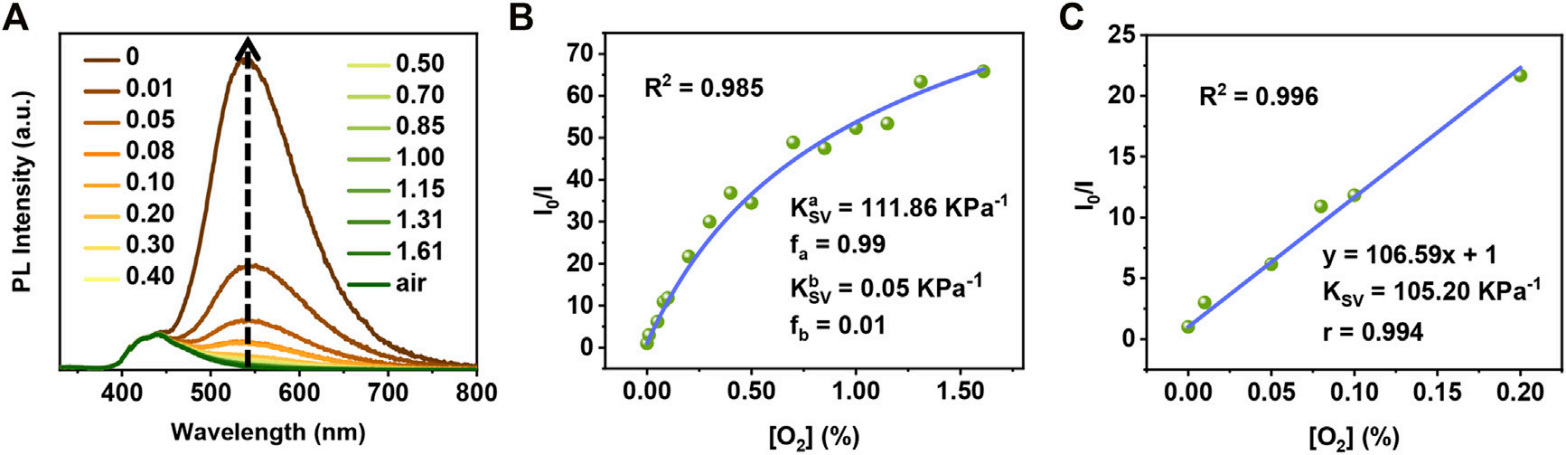
**(A)** Emission spectra of TA2P Zeonex® film at different oxygen concentrations. **(B)** The Demas model curve of TA2P Zeonex® film (fitting range: 0%–1.61%). **(C)** The Stern–Volmer curve of TA2P Zeonex® film (fitting range: 0%–0.20%).

Moreover, the PMMA polymer matrix was replaced with Zeonex^®^ for optimizing oxygen detection (Tomkeviciene et al., 2019), in which Zeonex^®^ is more oxygen-permeable and more rigid than PMMA. Due to the better oxygen permeability, the RTP of TA2P Zeonex^®^ film can be greatly quenched at a lower oxygen concentration, (Figure 4A). Different from PMMA matrix, the Stern–Volmer curve obtained based on *I*_0_/*I* with decreasing oxygen concentration shows an obvious non-linear change in the way of first flat and then steep, instead of a good linear fitting like that in PMMA matrix. Likewise, the Demas model was also used to fit the Stern–Volmer curve and to estimate *K*_SV_ (Figure 4B). As a result, its ${\text{K}}_{SV}^{a}$ is calculated as high as 111.86 KPa^−1^ (*f*_a_ = 0.99, an almost complete contribution to *K*_SV_). Such a high *K*_SV_ is very rare in oxygen-sensing materials, and what is more, the Stern–Volmer curve still exhibits excellent linearity when the oxygen concentration is below 0.20% (Figure 4C). Also, the *K*_SV_ was obtained as 104.62 KPa^−1^ by direct linear fitting according to the Stern–Volmer Eq. 1, which is very close to the fitting result using the Demas model. Such a high sensitivity provides a feasible alternative to use TA2P Zeonex^®^ film for an accurate detection of trace oxygen in the future (Borisov et al., 2011).

Relative to TA2P, TA1P shows an inferior performance in oxygen detection, and the detailed data is shown in Supplementary Figures S17, S18.

## 4 Conclusion

In summary, two phenyl-substituted derivatives of TA (TA1P and TA2P) were designed and synthesized for the efficient pure organic discrete-molecule RTP and the featured application of oxygen sensing and detection. These two compounds display the dual emission of fluorescence and RTP in discrete-molecule-doped film after deoxygenation, in which the efficient RTP is ascribed to the mechanism of folding-induced SOC enhancement. As a comparison, TA2P exhibits a higher RTP efficiency and more red-shifted RTP emission than those of TA1P, indicating a more significant electron conjugation effect between TA and phenyl group. Thus, TA2P has merits of both high efficiency of RTP and large spectral separation between dual emissions, which is more suitable for discrete-molecule ratiometric optical oxygen sensing and detection. As a result, TA2P demonstrates a Stern–Volmer quenching constant as high as 10.22 KPa^−1^ and the LOD can reach as low as 0.966 ppm in PMMA-doped film, which is one of the best results among oxygen-sensing materials. Additionally, PMMA was replaced with Zeonex® as a polymer matrix for further performance optimization. The Stern–Volmer constant of TA2P is estimated to be 111.86 KPa^−1^ in Zeonex^®^ matrix, which is a very high value. These results present a feasible way for the optimization design of pure organic discrete-molecule RTP materials based on TA derivatives, as well as provide a class of dual-emission materials for a promising application in ratiometric optical oxygen sensing and detection.

## Data Availability

The original contributions presented in the study are included in the article/Supplementary Material; further inquiries can be directed to the corresponding authors.
