# Supplementary material for: Efficient Room-Temperature Phosphorescence from Discrete Molecules Based on Thianthrene Derivatives for Oxygen Sensing and Detection
Source: Front Chem. 2022 Jan 27;9:810304. doi: 10.3389/fchem.2021.810304 (PMC8828495; doi:10.3389/fchem.2021.810304)
Supplement: Supplementary file 1 [file DataSheet1.pdf]

Supplementary Material

1 Supplementary experimental details

TA sample was purchased from J&k company and further purified by sublimation method.[1]

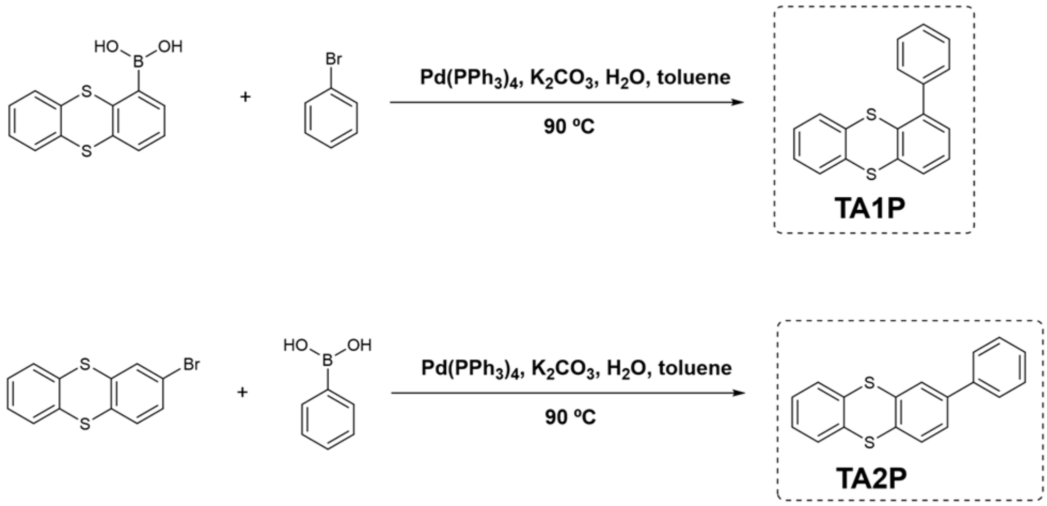

Scheme S1. Synthesis routes to TA1P and TA2P.

The synthesis of 1-phenylthianthrene (TA1P)

A mixture of **1-thianthrenylboronic acid** (520 mg, 2.00 mmol), **bromobenzene** (471 mg, 3.00 mmol), **K_2CO_3** (2.48 g, 18.00 mmol), 6 mL **distilled water** and 9 mL **toluene** was degassed and recharged with nitrogen. Then **$Pd(PPh_3)_4$** (69 mg, 0.06 mmol) was added in the mixture as catalyst, and the mixture was degassed and recharged with nitrogen again. After stirred and refluxed at 90 °C for 48 h under nitrogen atmosphere, the mixture was extracted with dichloromethane (DCM). The organic phase was dried with anhydrous sodium sulfate, filtered and concentrated in vacuum. It was purified via silica gel chromatography by the mixture of petroleum ether/DCM, and was recrystallized from DCM/methanol to give the product as white powder in 66% yield (385 mg). ^{1}H NMR (500 MHz, DMSO-d_6, 25 °C, TMS): δ = 7.62 (ddd, J = 16.5, 7.7, 1.4 Hz, 2H), 7.53 (dd, J = 8.0, 6.5 Hz, 2H), 7.50 – 7.44 (m, 1H), 7.48 – 7.39 (m, 4H), 7.39 – 7.32 (m, 2H), 7.28 (td, J = 7.5, 1.4 Hz, 1H); ^{13}C NMR (126 MHz, $CDCl_3$-*d*, 25 ℃, TMS): δ= 142.52 (C), 140.21 (C), 136.35 (C),

135.98 (C), 135.40 (C), 135.05 (C), 129.44 (CH), 129.28 (CH), 128.88 (CH), 128.52 (CH), 128.22 (CH), 128.15 (CH), 127.83 (CH), 127.77 (CH), 127.54 (CH), 127.06 (CH). GC-MS, EI, mass m/z: 292.42 [M^+]; Anal. calcd for $C_{12}H_{18}S_2$: C 73.94, H 4.14, S 21.93; found: C 73.97, H 4.20, S 21.61.

The synthesis of 2-phenylthianthrene (TA2P)

2-bromothianthrene was synthesized according to a previous report.[2] A mixture of **2-bromothianthrene** (590 mg, 2.00 mmol), **phenylboronic acid** (305 mg, 2.5 mmol), **K_2CO_3** (3.31 g, 24.00 mmol), 8 mL **distilled water** and 12 mL **toluene** was degassed and recharged with nitrogen. Then **$Pd(PPh_3)_4$** (69 mg, 0.06 mmol) was added in the mixture as catalyst, and the mixture was degassed and recharged with nitrogen again. After stirred and refluxed at 90 °C for 48 h under nitrogen atmosphere, the mixture was extracted with DCM. The organic phase was dried with anhydrous sodium sulfate, filtered and concentrated in vacuum. It was purified via silica gel chromatography by the mixture of petroleum ether/DCM, and was recrystallized from DCM/methanol to give the product as white powder in 72% yield (420 mg). ^{1}H NMR (500 MHz, DMSO-d_6, 25 °C, TMS): δ = 7.87 (d, J = 1.3 Hz, 1H), 7.74 – 7.69 (m, 2H), 7.66 (d, J = 1.2 Hz, 2H), 7.61 (ddd, J = 5.5, 3.3, 1.7 Hz, 2H), 7.48 (t, J = 7.6 Hz, 2H), 7.39 (ddd, J = 13.1, 6.6, 2.3 Hz, 3H); ^{13}C NMR (126 MHz, $CDCl_3$-*d*, 25 °C, TMS): δ = 141.09 (C), 139.69 (C), 136.14 (C), 135.57 (C), 135.46 (C), 134.39 (C), 128.91 (CH), 128.81 (CH), 128.76 (CH), 127.75 (CH), 127.28 (CH), 127.00 (CH), 126.51 (CH). GC-MS, EI, mass m/z: 292.42 [M^+]; Anal. calcd for $C_{12}H_{18}S_2$: C 73.94, H 4.14, S 21.93; found: C 74.22, H 4.04, S 21.02.

2 Supplementary figures and schemes

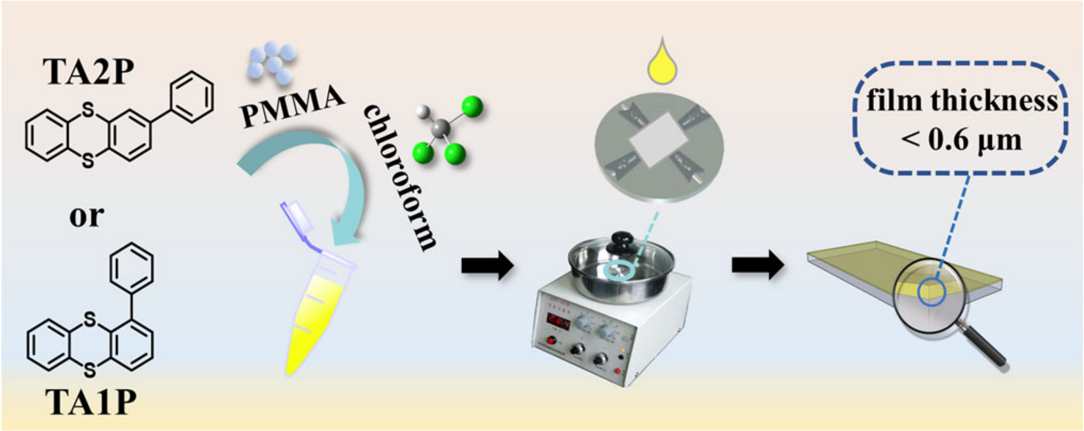

Scheme S2. Schematic diagram of preparation process of the films for oxygen sensing and detection.

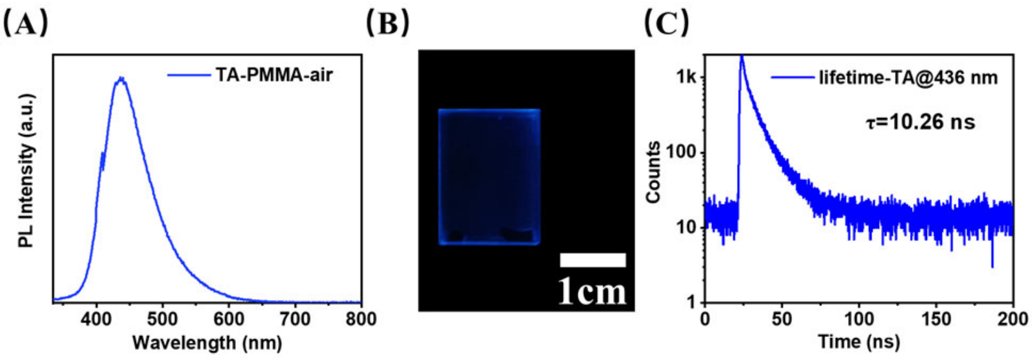

Figure S1. **(A)** Emission spectra, **(B)** image and **(C)** time-resolved emission spectra of TA polymethyl methacrylate (PMMA) film in the air.

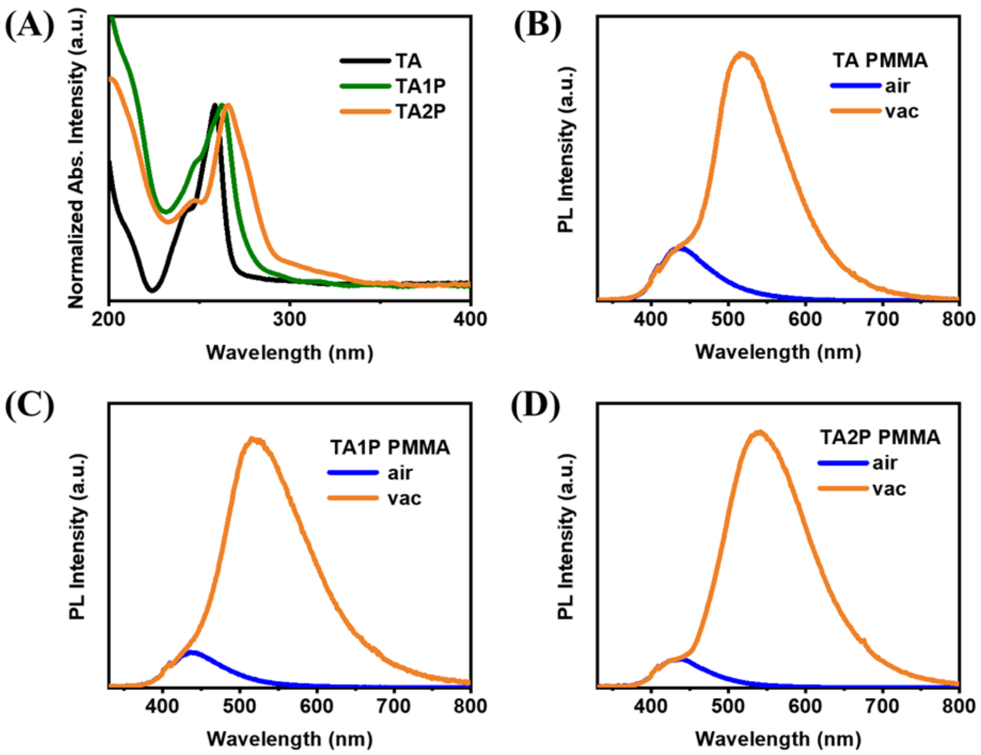

Figure S2. (A) UV-vis absorption spectra of TA, TA1P and TA2P PMMA films. **(B) (C) (D)** Emission spectra of TA, TA1P and TA2P PMMA films in the air (fluorescence) and in a vacuum (room-temperature phosphorescence, RTP).

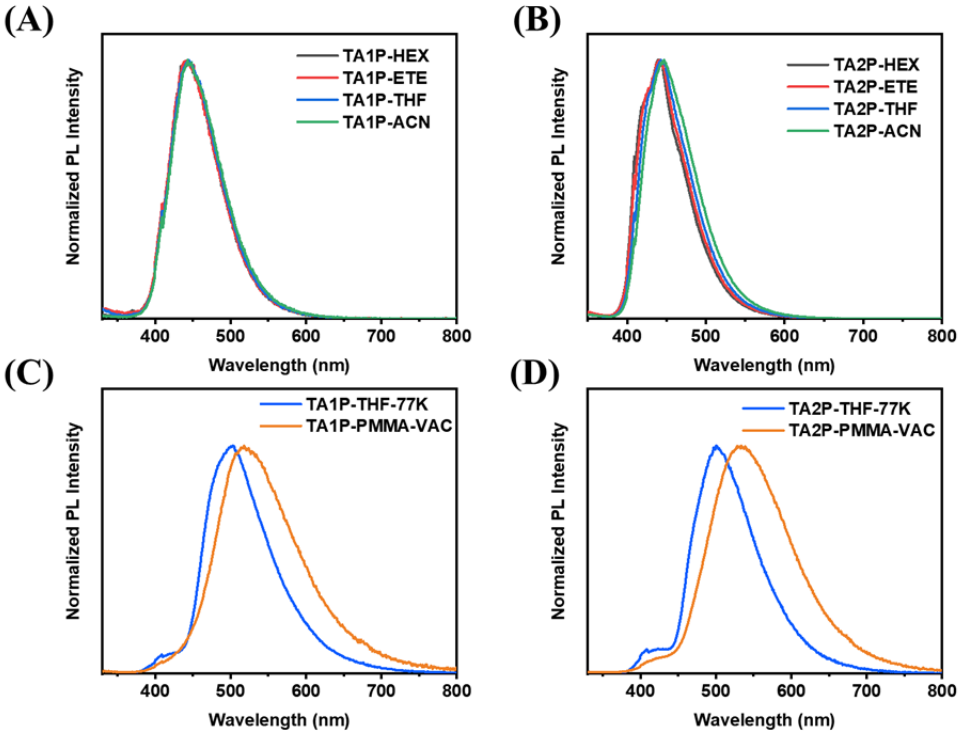

Figure S3. (A) (B) Emission spectra of TA1P and TA2P in dilute solutions (10^{-5} mol/L) at room temperature (RT). Here, HEX= n-Hexane, ETE= Ethyl ether, THF= Tetrahydrofuran, ACN= Acetonitrile. **(C) (D)** Comparison of the emission spectra of TA1P and TA2P PMMA films under vacuum with their THF dilute solutions (10^{-5} mol/L) at 77K

As shown in **Figure S3**, as for TA1P or TA2P, the emission spectrum of the dilute solution is consistent with that of the doped PMMA film, proving that the emission properties of the film can be attributed to the dispersed molecules. As for TA1P and TA2P, their THF solutions at 77 K demonstrate the slightly blue-shifted phosphorescence emission relative to their doped PMMA films, indicating the restricted geometrical relaxation from folded conformation to planar one at 77 K.

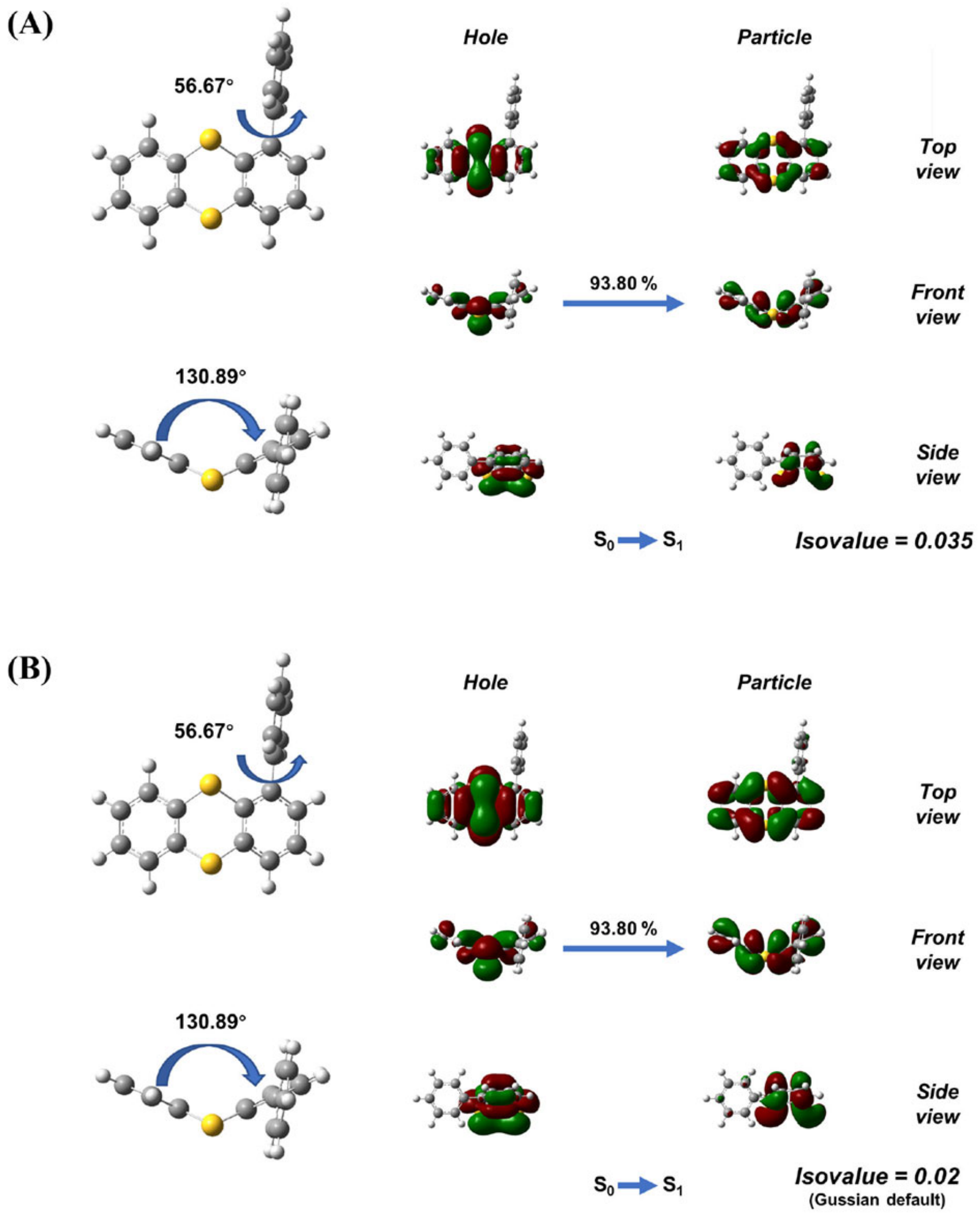

Figure S4. Molecular conformations (ground state) of TA1P by theoretical optimization and the NTOs of the S_1 state when the Isovalue value is **(A)** 0.035 and **(B)** 0.02.

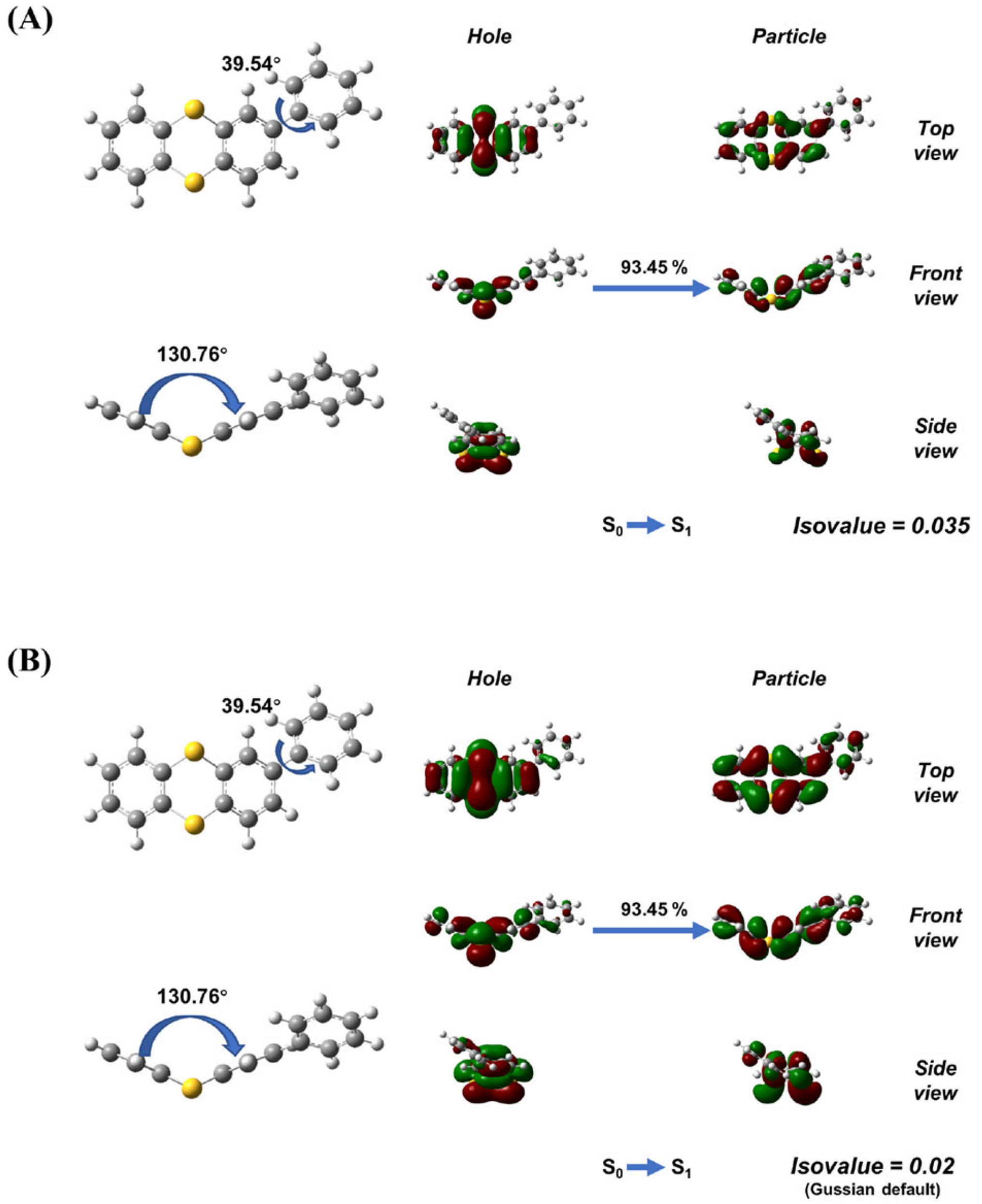

Figure S5. Molecular conformations (ground state) of TA2P by theoretical optimization and the NTOs of the S_1 state when the Isovalue value is **(A)** 0.035 and **(B)** 0.02.

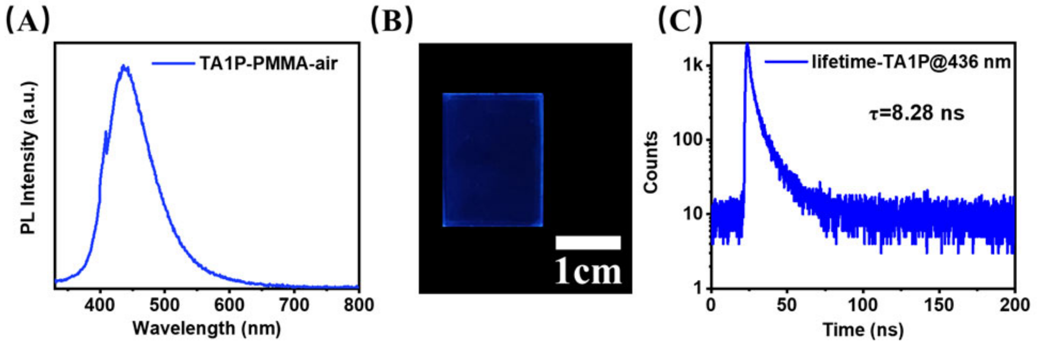

Figure S6. (A) Emission spectra, **(B)** image and **(C)** time-resolved emission spectra of TA1P PMMA film in the air.

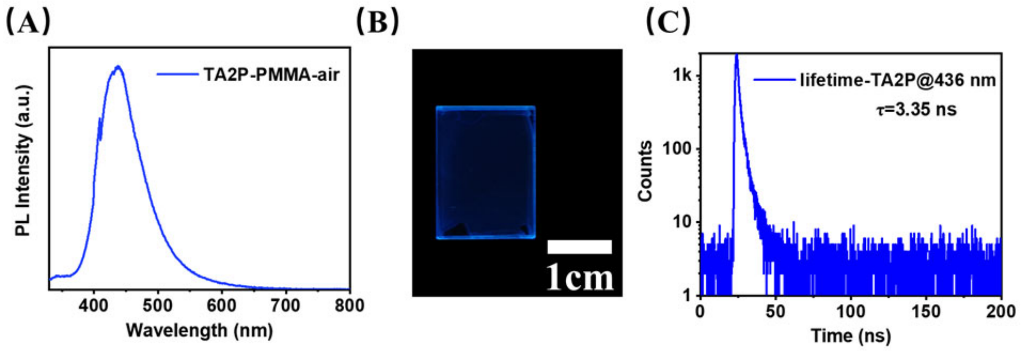

Figure S7. (A) Emission spectra, **(B)** image and **(C)** time-resolved emission spectra of TA2P PMMA film in the air.

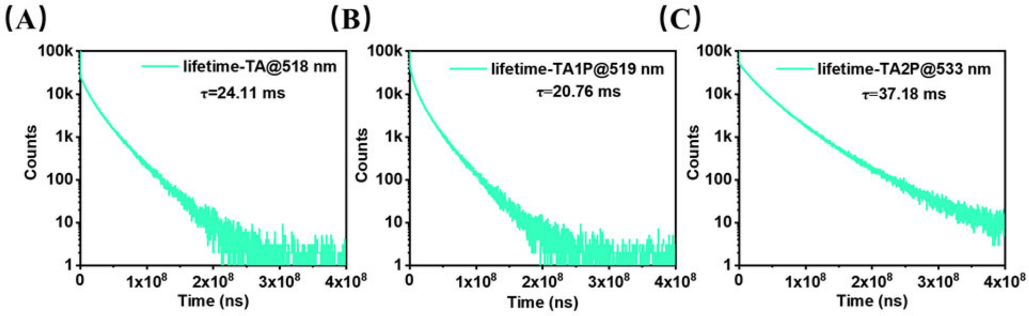

Figure S8. Time-resolved emission spectra of **(A)** TA PMMA, **(B)** TA1P PMMA, and **(C)** TA2P PMMA at long wavelengths in a vacuum.

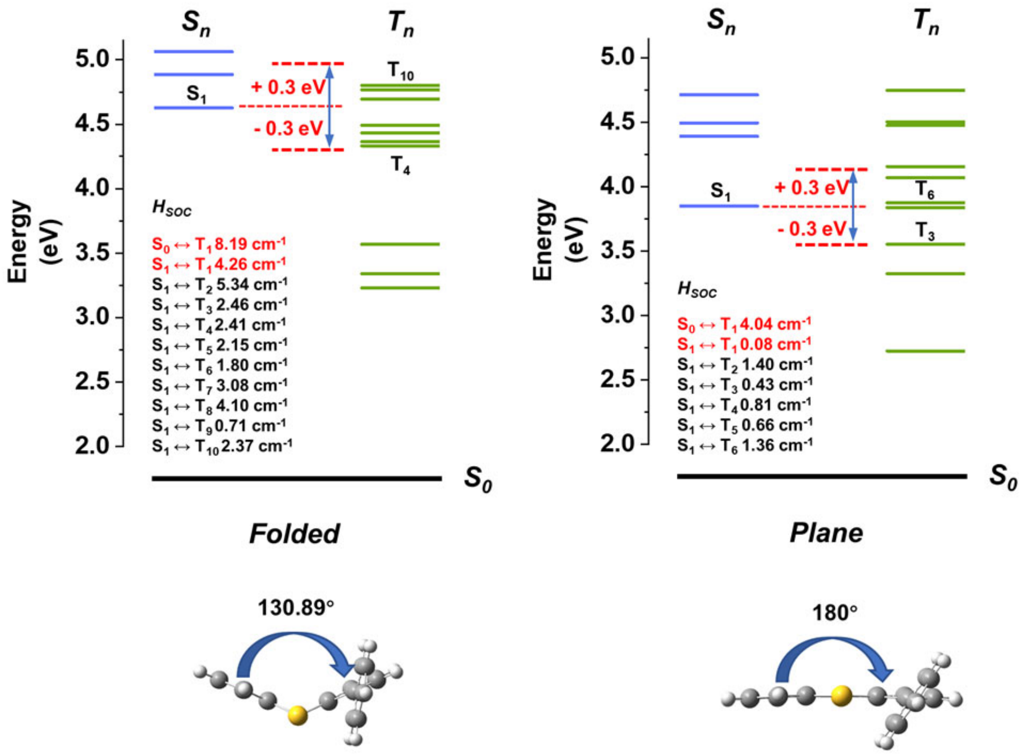

Figure S9. Energy levels of TA1P and SOC coefficients between different energy levels for (A) the folded conformation (ground state) by optimization method and (B) the planar conformation (the dihedral angle is 180°) by artificial adjustment.

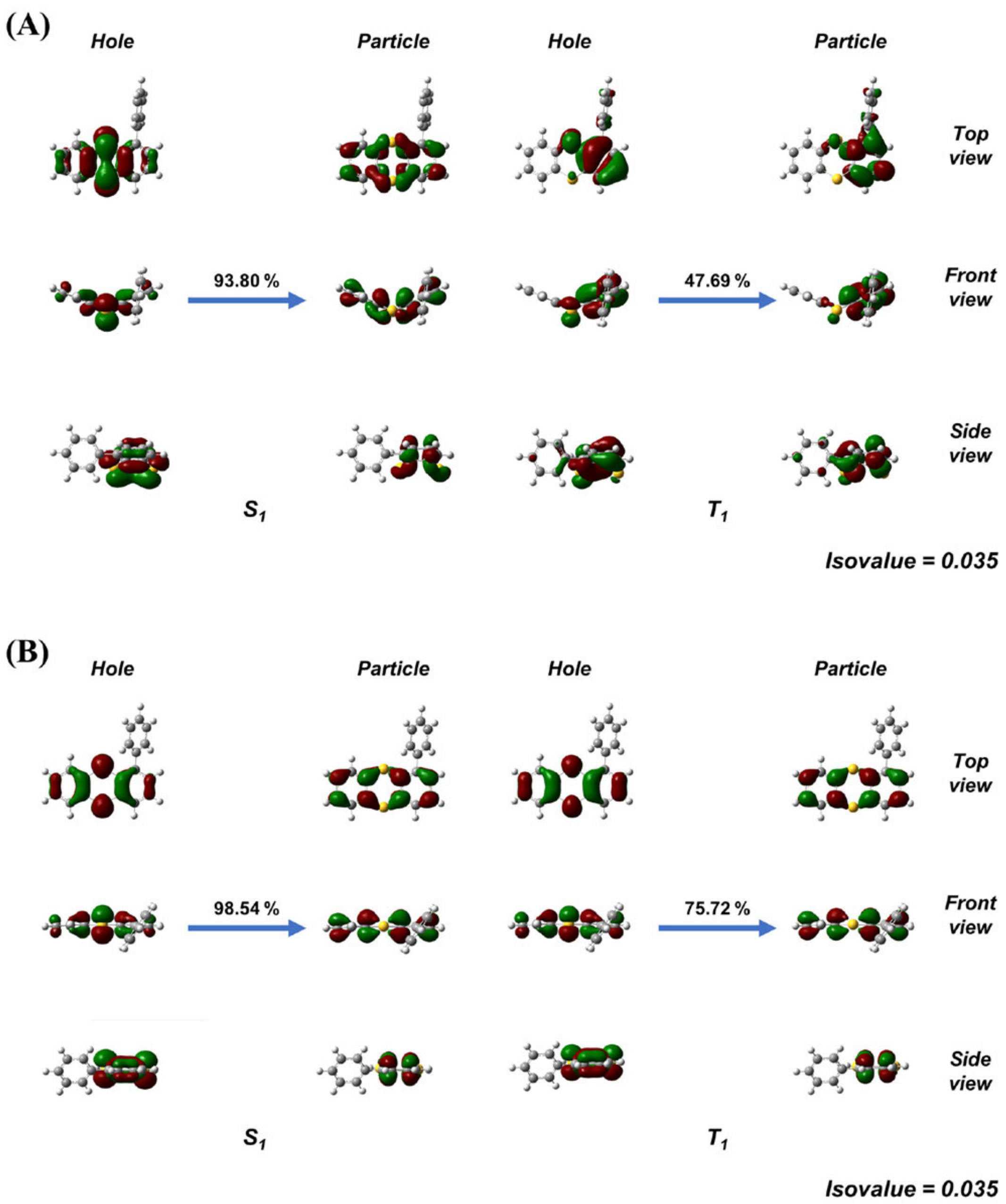

Figure S10. NTOs of the S_1 and T_1 states of TA1P for (A) the folded conformation (ground state) by optimization method and (B) the planar conformation (the dihedral angle is 180°) by artificial adjustment.

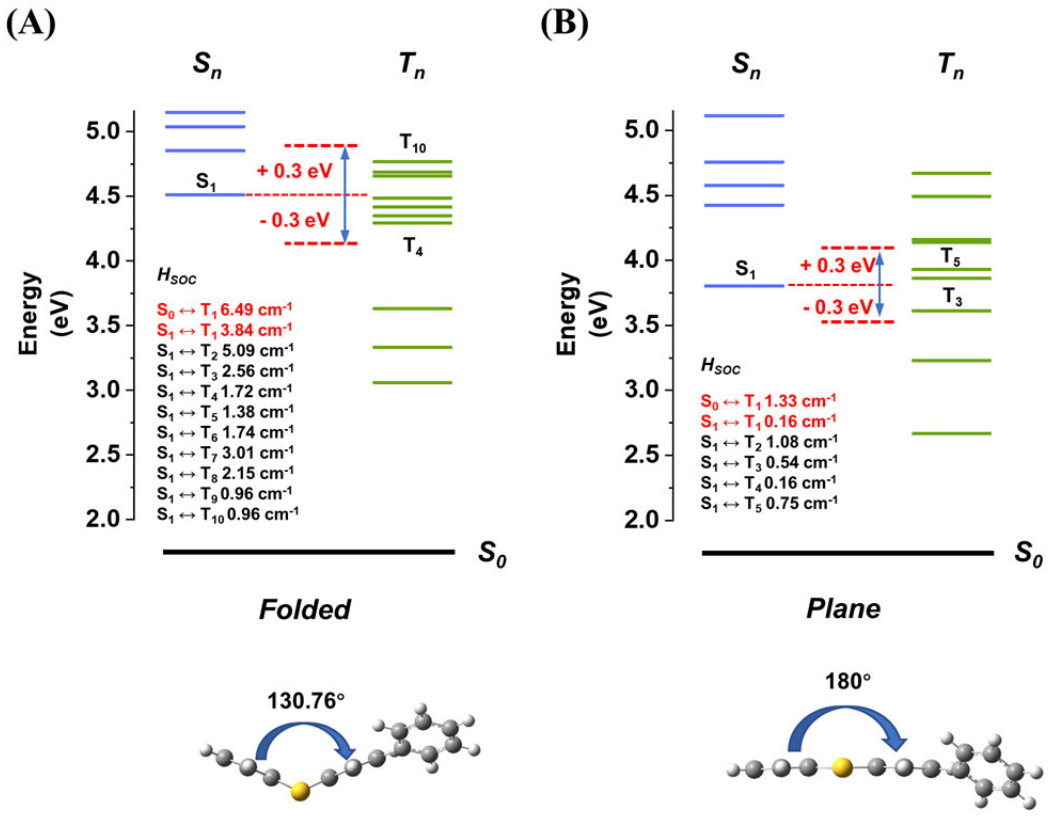

Figure S11. Energy levels of TA2P and SOC coefficients between different energy levels for (A) the folded conformation (ground state) by optimization method and (B) the planar conformation (the dihedral angle is 180°) by artificial adjustment.

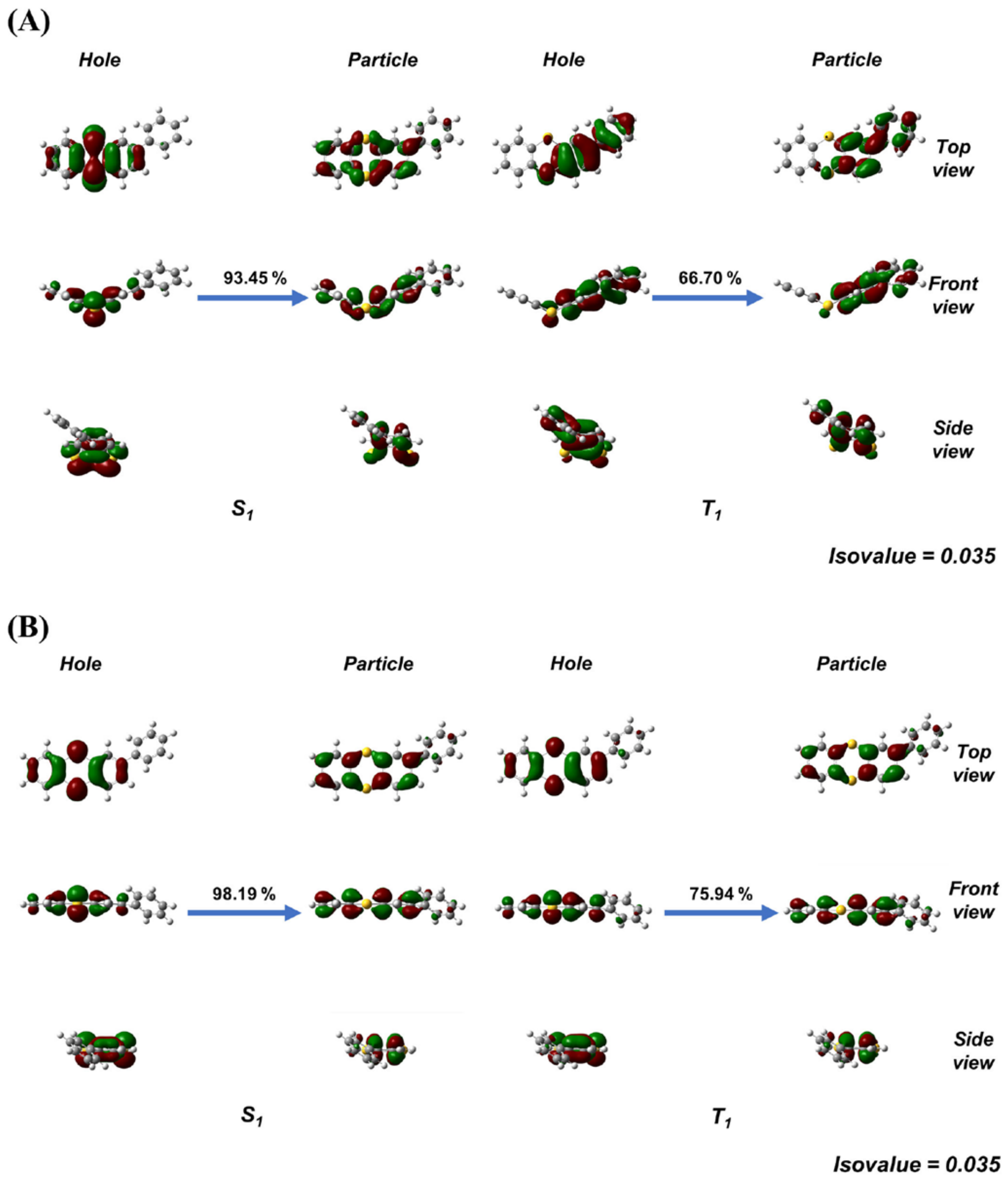

Figure S12. NTOs of the S_1 state and T_1 state of TA2P for (A) the folded conformation (ground state) by optimization method and (B) the planar conformation (the dihedral angle is 180°) by artificial adjustment.

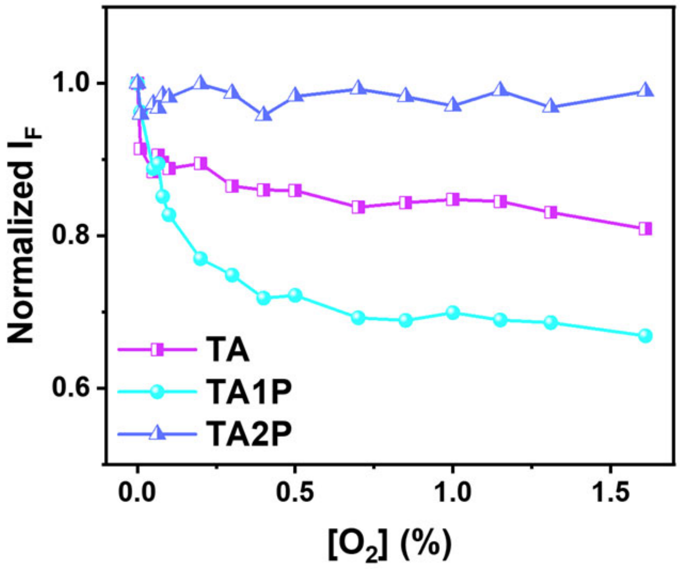

Figure S13. Plot of the fluorescence intensity (I_F) against the oxygen concentration for the three PMMA films (the I_F in a vacuum is defined as unity).

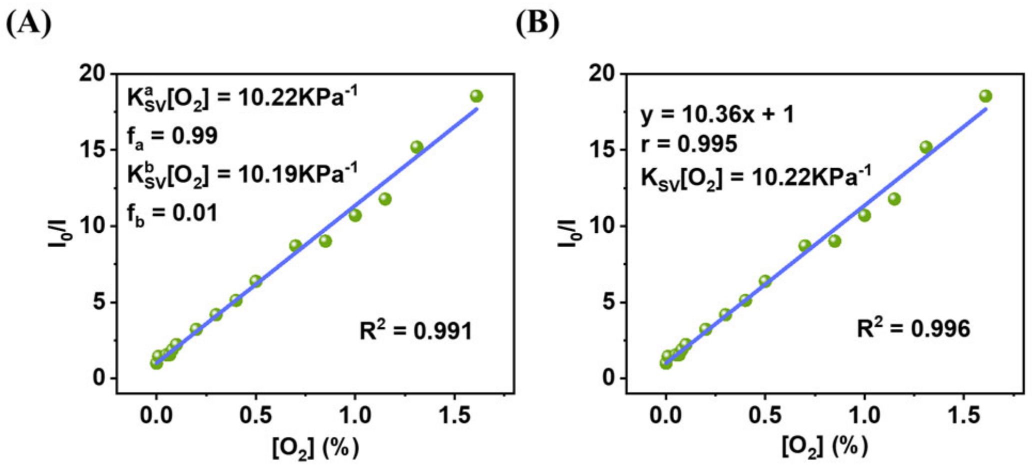

Figure S14. Comparison of **(A)** the "two-site model" by Demas and co-workers and (**B**) linear fitting result based on the emission spectrum method of TA2P PMMA film. The "Demas model" was also used to fit the Stern–Volmer curve based on I_0/I, and the K_{SV} value is almost the same as that of the linear fitting, confirming the reliability of the linear fitting of Stern–Volmer equation.

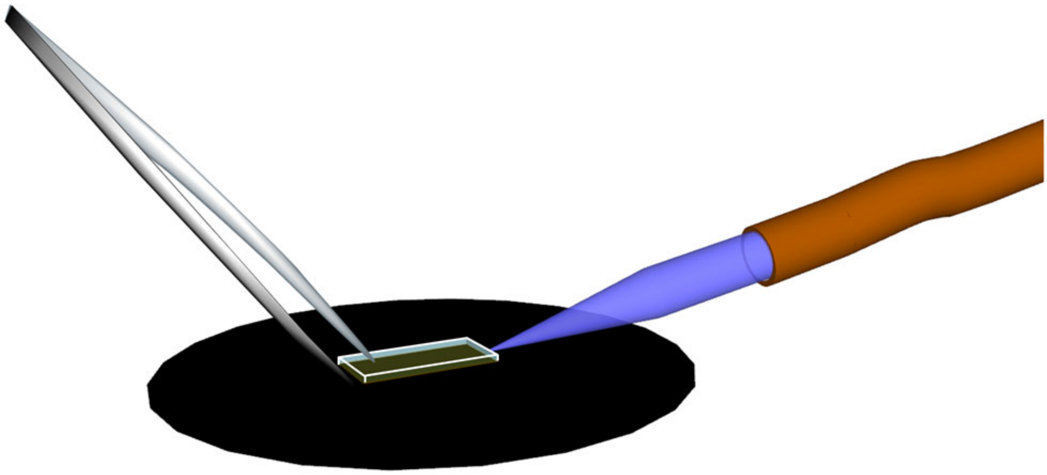

Figure S15. Schematic diagram of the home-made device for real-time detection of oxygen concentration in a nitrogen-oxygen mixture.

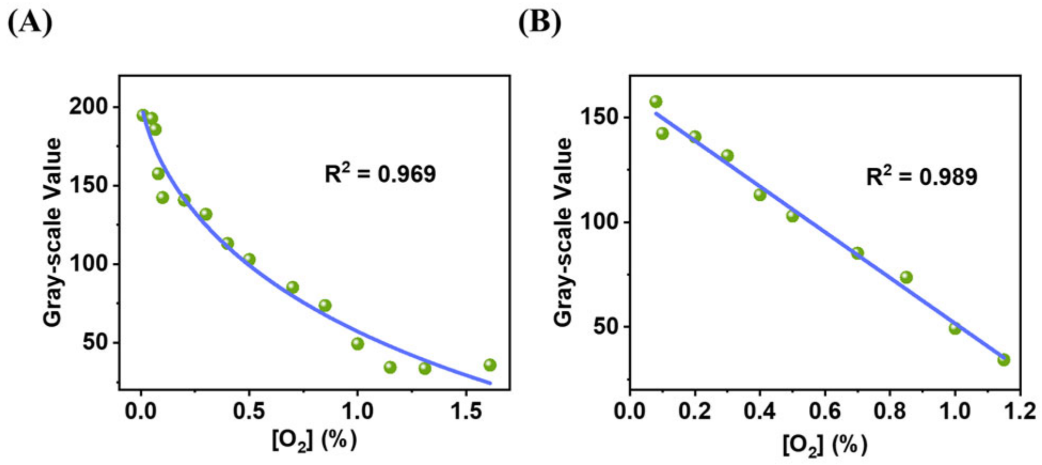

Figure S16. Plot of the gray-scale value against oxygen concentration for TA2P PMMA film in the fitting range: (A) 0.01~1.61 %; (B) 0.08~1.15%.

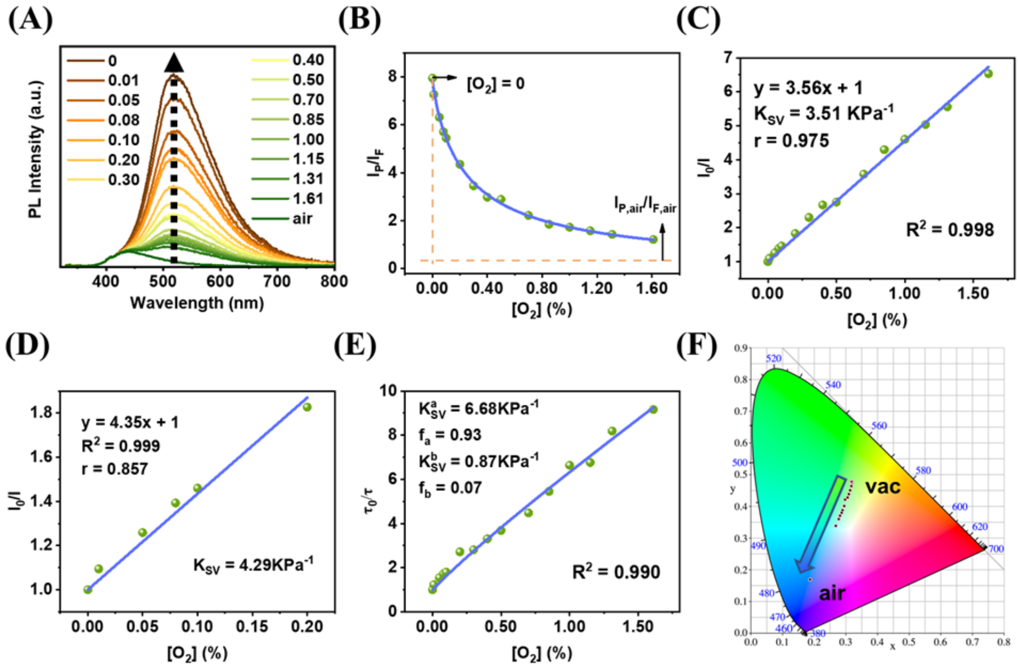

Figure S17. (A) Emission spectra of TA1P PMMA film at different oxygen concentrations (excited by 300 nm). **(B)** The trend of I_P/I_F with increasing oxygen concentration for TA1P PMMA film. **(C)** Stern–Volmer curve of TA1P PMMA film (fitting range: 0~1.61%). **(D)** Stern–Volmer curve of TA1P PMMA film (fitting range: 0~0.20%). **(E)** "Demas model" curve based on lifetimes of TA1P PMMA film. **(F)** CIE coordinates of TA1P PMMA film with varying oxygen concentration.

TA1P also have high sensitivity in oxygen detection, but the observed fluorescence intensity of TA1P is actually affected by the RTP intensity at low oxygen concentrations, that is, the reference signal loses its calibration ability. This case adversely affects the judgment of the oxygen detection performance of materials. Alternatively, the Stern–Volmer curve obtained by the lifetime measurement is more meaningful for TA1P (**Figure S18**), because the RTP lifetime will not be affected by other conditions except for the oxygen concentration in the test environment.

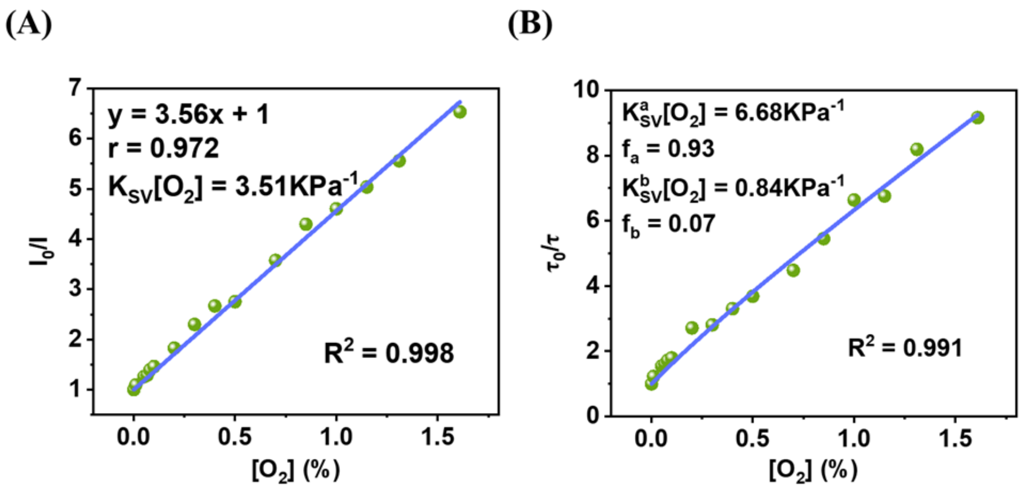

Figure S18. (**A**) Stern–Volmer curve of TA1P PMMA film based on the emission spectrum method. (**B**) "Demas model" curve of TA1P PMMA film based on the lifetime method.

3 Supplementary references

[1] Liu, H., Gao, Y., Cao, J., Li, T., Wen, Y., Ge, Y., et al. (2018). Efficient room-temperature phosphorescence based on a pure organic sulfur-containing heterocycle: folding-induced spin-orbit coupling enhancement. *Mater. Chem. Front.* 2(10), 1853-1858. doi: 10.1039/c8qm00320c.

[2] Liu, H., Yao, L., Li, B., Chen, X., Gao, Y., Zhang, S., et al. (2016). Excimer-induced high-efficiency fluorescence due to pairwise anthracene stacking in a crystal with long lifetime. *Chem. Commun.* 52(46), 7356-7359. doi: 10.1039/C6CC01993E.
